# Response to major peach and peanut allergens in a population of children allergic to peach

**DOI:** 10.1186/2045-7022-5-S3-P129

**Published:** 2015-03-30

**Authors:** Natalia Blanca-Lopez, Ana Aranda, Alejandra Garcia-Blanca, Diana Perez, Maria Luisa Somoza, Francisca Gomez, Cistobalina Mayorga, Maria Jose Torres, Araceli Diaz Perales, Miguel Blanca, Gabriela Canto

**Affiliations:** 1 Allergy Service, Infanta Leonor Hospital, Madrid, Spain; 2 Research Laboratory, IBIMA, Regional University Hospital of Malaga, UMA, Malaga, Spain; 3 Allergy Service, Carlos Haya Hospital, Malaga, Spain; 4 Research Laboratory, Carlos Haya Hospital-FIMABIS, Malaga, Spain; 5 Centre for Plant Biotechnology and Genomic, Polytechnical University of Madrid, Madrid, Spain

## Introduction

Peach allergy is one of the most common allergies in subjects with reactions to plant-foods. The relationship between peach allergy and other foods and the sensitization pattern in these patients have not been studied in detail. We study the sensitization to relevant allergens, such as peach and peanut LTP, Prup3 and Arah9, and seed storage protein, Arah2. Studies were carried out in child/adolescent population from 1-20 y.o allergic to peach, in order to evaluate the relationship between peach and peanut allergy.

## Methods

Children and adolescents allergic to peach were chosen from a large number of patients with allergy to plant-foods. They were classified in: A) those allergic to peach with tolerance to peanut and B) those allergic to peach and peanut. The IgE response was measured by ImmunoCAP to Arah2, Arah9 and Prup3 and the relationship with the different clinical entities as well as the variation according to age was analyzed.

## Results

From a total of 348 subjects evaluated, 39% were allergic to peach. The median age was 11.60 years and 82% had sensitization to pollens, with *Phleum*, *Olea*, *Platanus* and *Artemisia* the most relevant. Urticaria appeared in 53%, followed by oral allergy syndrome (36%) and anaphylaxis (9%). Specific IgE to Prup3, Arah9 and Arah2 were detected in 58%, 51% and 12% of patients allergic to peach, respectively. From peach allergic patients, 75% reported symptoms only with peach and 25% to peach and peanut. Comparison between groups A and B showed no significant differences in clinical entities and characteristics analyzed. IgE response to Prup3 was similar for both groups, and Arah9 (45% vs. 66%) and Arah2 (11% vs. 22%) were slightly greater in group B but not significant. An analysis of positivity to these allergens did not show significant differences due to age.

## Conclusions

Peach allergy is very frequent in subjects with allergy to plant food with Prup3 the relevant protein in the Mediterranean area. Among the subjects allergic to peach there is a high proportion of patients also allergic to peanut and whose primary sensitization pattern appears to be due to LTPs.

